# Supplementary figures and images for: Quantification of malaria antigens PfHRP2 and pLDH by quantitative suspension array technology in whole blood, dried blood spot and plasma
Source: Malar J. 2020 Jan 9;19:12. doi: 10.1186/s12936-019-3083-5 (PMC6953214; doi:10.1186/s12936-019-3083-5)

**A**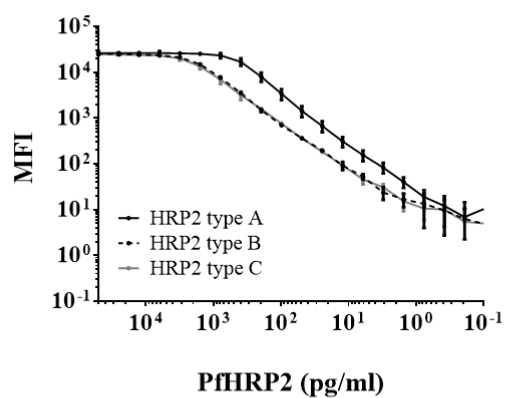**B**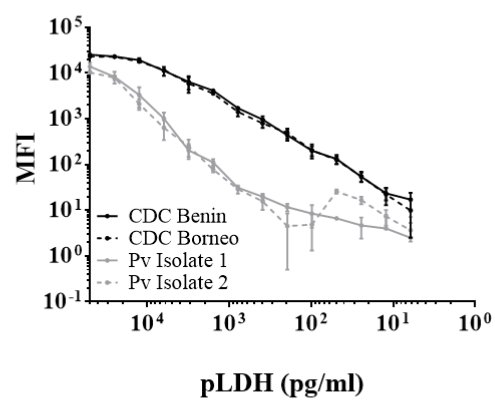**C**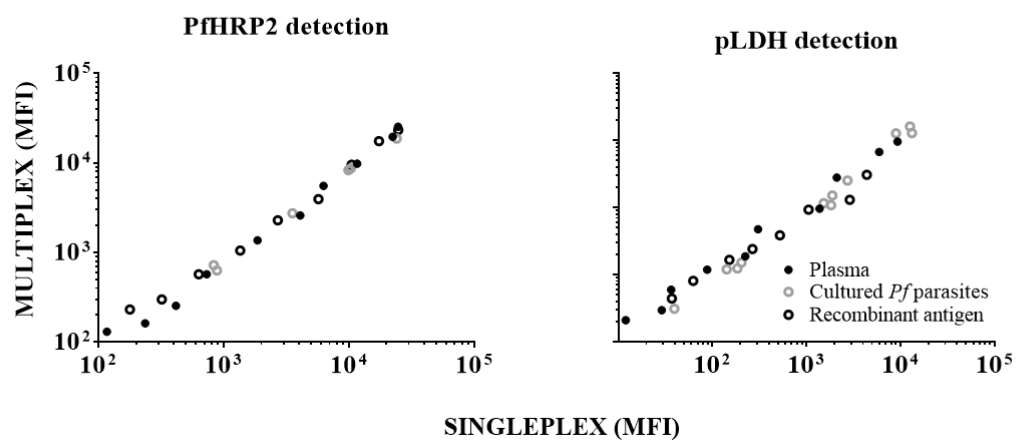

Supplement: Supplementary file 2 — Additional file 2: Figure S1. Assay optimization. (A) Serial dilutions of recombinant PfHRP2 types A, B and C were assayed to determine the lowest concentration at which each antigen is detected; (B) P. falciparum Benin I and Borneo and P. vivax field isolates were assayed in a serial dilution fashion to assess differences between the analytical sensitivity for P. falciparum and P. vivax pLDH; (C) PfHRP2 and pLDH positive samples (plasma, cultured field isolates and recombinant proteins) were assayed in singleplex (X axes) and multiplex (Y axes). [file 12936_2019_3083_MOESM2_ESM.pdf]
